# Analysis of Dip2B Expression in Adult Mouse Tissues Using the LacZ Reporter Gene

**DOI:** 10.3390/cimb43020040

**Published:** 2021-06-30

**Authors:** Rajiv Kumar Sah, Noor Bahadar, Fatoumata Binta Bah, Salah Adlat, Zin Mar Oo, Luqing Zhang, Fawad Ali, M S Zobaer, Xuechao Feng, Yaowu Zheng

**Affiliations:** 1 Key Laboratory of Molecular Epigenetics, Institute of Genetics and Cytology, Northeast Normal University, Changchun 130024, China; kum235@nenu.edu.cn (R.K.S.); noor100@nenu.edu.cn (N.B.); cissehbah@yahoo.fr (F.B.B.); land788@nenu.edu.cn (S.A.); cengy696@nenu.edu.cn (Z.M.O.); zhanglq479@nenu.edu.cn (L.Z.); 2 WISH Biotechnologies, Beihu Scinece Park B, Changchun 130000, China; 3 Department of Chemistry, Bacha Khan University, Charsadda 6431, KP, Pakistan; fawadchemist@bkuc.edu.pk; 4 McGovern Medical School, The University of Texas Health Science Center at Houston (UTHealth), Houston, TX 77030, USA; M.S.Zobaer@uth.tmc.edu

**Keywords:** *Dip2b*, LacZ, nervous system, reproductive system, vascular system, respiratory system

## Abstract

Disconnected (disco)-interacting protein 2 homolog B (Dip2B) is a member of the Dip2 superfamily and plays an essential role in axonal outgrowth during embryogenesis. In adults, Dip2B is highly expressed in different brain regions, as shown by in situ analysis, and may have a role in axon guidance. However, the expression and biological role of Dip2B in other somatic tissues remain unknown. To better visualize Dip2B expression and to provide insight into the roles of Dip2B during postnatal development, we used a *Dip2b^tm1a(wtsi)komp^* knock-in mouse model, in which a LacZ-Neo fusion protein is expressed under *Dip2b* promoter and allowed Dip2B expression to be analyzed by X-gal staining. qPCR analyses showed that *Dip2b* mRNA was expressed in a variety of somatic tissues, including lung and kidney, in addition to brain. LacZ staining indicated that Dip2B is broadly expressed in neuronal, reproductive, and vascular tissues as well as in the kidneys, heart, liver, and lungs. Moreover, neurons and epithelial cells showed rich staining. The broad and intense patterns of Dip2B expression in adult mice provide evidence of the distribution of Dip2B in multiple locations and, thereby, its implication in numerous physiological roles.

## 1. Introduction

Disco-interacting protein 2 homologs (Dip2) have been identified in numerous species and are highly conserved from prokaryotes to eukaryotes [1]. In humans, the DIP2 family protein consists of three members, namely, DIP2A, DIP2B, and DIP2C located on chromosomes 12q13.12, 21q22.3, and 10p15.3, respectively. Bioinformatic analysis has suggested that mammalian DIP2s (i.e., DIP2A, DIP2B, and DIP2C) contain three putative and conserved functional domains, namely one DNA methyltransferase 1-associated protein 1 (DMAP1)-binding domain (14–131 aa) and two adenosine monophosphate (AMP)-binding domains (370–822 and 1025–1495 aa) with similar roles in acetyl co-enzyme synthesis [2]. The presence of these domains also suggests that the encoded protein may participate in DNA methylation [3]. The mouse Dip2 gene has three homologs, *Dip2A*, *Dip2B*, and *Dip2C*, distributed on chromosomes 10, 15, and 13, respectively [2]. The mouse *Dip2b* gene was first isolated from the mouse embryonic E12.5 cDNA library [1]. The *Dip2b* locus consists of 38 exons spanning a region of 200.81 kb on chromosome 15 (NCBI. Available online: https://www.ncbi.nlm.nih.gov/gene/239667 (23 June 2021); Supplementary File S1). The exon region encodes a putative protein of 1574 amino acids. Percentage similarities, as calculated by the program Align (EBI), show that the predicted mouse Dip2B protein shares a 96.1% overall identity with the human DIP2B sequence [3].

The *Dip2* gene was first identified and studied in *Drosophila*. Dip2 is a novel transcription factor that binds *Disconnected* protein (*Disco*) and supports proper neuronal connection during larva visual system development [4,5,6]. *Drosophila* *Dip2* is expressed in brain lobes and the ventral cord during embryogenesis and regulates axonal bifurcation of mushroom body neurons through the regulation of c-Jun N-terminal kinase [7,8]. In *Caenorhabditis elegans*, Dip2 is expressed in most neurons, including VNC motor neurons, HSN, PVD, and mechanosensory neurons, as well as in epidermal cells. It is known that Dip2 functions in maintaining neuronal morphology and axon regrowth after injury [9].

In humans, Northern blot analysis revealed that DIP2B is expressed in the brain, placenta, skeletal muscle, heart, kidneys, pancreas, lungs, spleen, and colon and may be associated with neurocognitive problems linked with the fragile site FRA12a on chromosome 12q13.1 [3]. Several bioinformatics studies have also discovered associations between DIP2B and numerous diseases, including schizophrenia, coronary artery disease (CAD), cervical squamous cell carcinoma, and colorectal cancer [10,11,12,13]. In mice, *mDip2* mRNA remains specific to the developing nervous system and may provide positional cues required for axon pathfinding and patterning [1]. According to the International Knockout Mice Consortium (IKMC) program, *Dip2b* knockout decreases circulating free fatty acid and leukocyte numbers [14,15]. Although a previous report produced evidence for the expression of the *Dip2* gene in mice using Northern blot analysis, until now, validated tools and protocols for in situ *Dip2b* expression analysis in mice using the LacZ (β-galactosidase) reporter have not been available. In addition, in vivo analysis of *Dip2b* gene expression in non-neuronal cell types remains unknown. Notably, the physiopathological roles of DIP2B in mammals are still far from clear.

Recently, we reported that Dip2B is widely expressed during embryogenesis and its loss leads to prenatal lethality, possibly due to abnormal lung development [16]. In the present study, we aimed to delineate, in detail, the tissues that may require Dip2B in adults by characterizing and analyzing the expression of Dip2B using *Dip2b^tm1a(wtsi)komp^* (*Dip2b^Tm1a^*) knock-in mice generated by the Knockout Mouse Project (KOMP) [17]. Transgenic expression of LacZ can be easily detected by enzyme histochemical staining using chromogenic substrates such as 5-bromo-4-chloro-3-indolyl-β-D-galactosidase (X-gal), 5-bromo-indolyl-β-O-galactopyranoside (Bluo-gal), and derivatives thereof. β-Galactosidase hydrolyzes X-gal to form a highly sensitive blue precipitate [18,19,20,21,22,23,24]. Subsequently, reporter gene approaches involved the detection of LacZ, another enzyme, or fluorescent proteins, have been used in many studies to characterize gene expression patterns in animals. The LacZ reporter is particularly useful since it requires no special imaging equipment and requires only standard histochemical staining methods. The whole-mount and frozen sections of *Dip2b^Tm1a^* knock-in mice were stained with X-gal, in parallel with wild-type littermate controls.

## 2. Materials and Methods

### 2.1. Animals

*Dip2b^tm1a (KOMP)wtsi^* (*Dip2b^tm1a/+^*) mice were purchased from the Knockout Mice Project (KOMP), while C57BL/6J mice were purchased from Vital River (Beijing, China). All mice were kept in a clean facility in Northeast Normal University. All animals were kept in IVC cages with free access to water and food at 20 °C and 50 ± 20% relative humidity under a 12/12 h light/dark cycle and pathogen-free conditions. All experimental procedures were conducted based on the Guide for Care and Use of Laboratory Animals of National Institutes of Health and approved by the Institutional Animal Care and Use Committee of Northeast Normal University (NENU/IACUC, AP2013011). The mice were sacrificed using 1% pentobarbital anesthesia at a dose of 10 mg/kg. Three biological replicates for *Dip2b^tm1a/+^* and wild-type littermates of the same age were used in every experiment.

### 2.2. Genotyping

Genomic DNA from the adult mice was extracted from their tail tips and genotyped by PCR as previously described. Briefly, a tail sample was digested in GNTK-Proteinase K solution at 55–60 °C overnight and lysate boiled for 15 min, centrifuged at 1200 rpm, and subjected to PCR using the following conditions: 94 °C for 2 min; followed by 30 cycles of denaturation at 94 °C for 30 s, annealing at 60 °C for 30 s, and extension at 72 °C for 30 s; and final extension at 72 °C for 5 min, then held at 4 °C. PCR products were electrophoresed on 0.8% agarose gels. The nucleotide sequences of the PCR primers for genotyping were as follows: tm1a-F-TGAGACTGAGCTTGGCTACCACA and tm1a-R-TCCTCC TACATAGTTGGCAGTGT, and wt-F-AGTTAAGGCTGAGCATGGTGGGA and wt-R-TAGGGCTCTCACAGATCAGAGCT.

### 2.3. LacZ Staining of Whole-Mount and Frozen Sections

All tissues were harvested from 8-week-old *Dip2b^tm1a/+^* and *Dip2b^+/+^* (WT) mice. Mice were anesthetized with pentobarbital and then perfused with 4% PFA. The tissues were immediately dissected and placed in cold PBS, followed by fixation in 2% PFA, 0.25% glutaraldehyde, and 0.01% NP40 in PBS for 2 h with agitation at 4 °C. The tissues were then washed in rinsed buffer (2 mM MgCl_2_, 0.02% NP40, and 0.01% Na-deoxycholate in PBS) and processed for whole-mount X-Gal staining (30 mM K_3_Fe(CN)_6_, 30 mM K_4_Fe(CN)_6_3H_2_O, 2 mM MgCl_2_, 0.01% Na-deoxycholate, 0.02% NP40, and 1 mg/mL 5-bromo-4-chloro-3-indolyl-β-D-galactopyranoside) at 37 °C for 6–8 h or until the desired intensity was observed. Stained tissues were then washed in PBS, postfixed in 4% PFA, and stored in 70% glycerol at 4 °C.

For frozen section X-gal staining, the tissues were incubated in 20% sucrose overnight at 4 °C and then embedded in an OCT compound (Tissue-Tek, Torrance, CA, USA) and frozen at −80 °C. Serial cryosections of 18 μm thickness were cut and collected onto Superfrost^TM^ plus microscope slides. Sections were then dried on a slide warmer, fixed with 2% PFA for 10 min, and washed in rinsing buffer. X-gal staining was performed in a dark chamber at 37 °C overnight or until the desired staining was observed. After staining, the sections were fixed in 4% PFA for 5 min and washed in PBS. The sections were counter-stained in 0.25% eosin solution. Whole-mount and frozen section images were taken using an Olympus microscope (SXL-ILLB2-200, Tokyo, Japan) and a Canon digital camera (DSI26431, Tokyo, Japan).

### 2.4. RNA Isolation and Real-Time PCR

Total RNA from different tissues of 8-week-old male CBL57/6 mice was extracted using TRIzol reagent (Invitrogen, Waltham, MA, USA) according to the manufacturer’s instructions. Total RNA (1 μg) was reverse transcribed using a Primescript^TM^ II cDNA Synthesis Kit (Takara Biotechnology, Dalian, China). Quantitative real-time PCR was performed using a Light Cycler 480 sequence detection system (Roche, Indianapolis, IN, USA) and SYBR II premix (Takara). All results were normalized to 18S ribosomal RNA, and relative quantification was calculated using comparative threshold cycle (ΔΔCt) values for each biological replicate. *Dip2b* primers (mDip2b-QPCRF: TCTGGAGGTGCGAGAGATGA; mDip2b-QPCRR: TTGAGCGGTTGATCCAGGAC) and 18S primers (18S F: CGCCGCTAGAGGTGAAATTC; 18S R: CGAACCTCCGACTTTCGTTCT) were used for amplification.

## 3. Results

### 3.1. Genotyping of Dip2b^tm1a^ Mice

**Figure 1:**
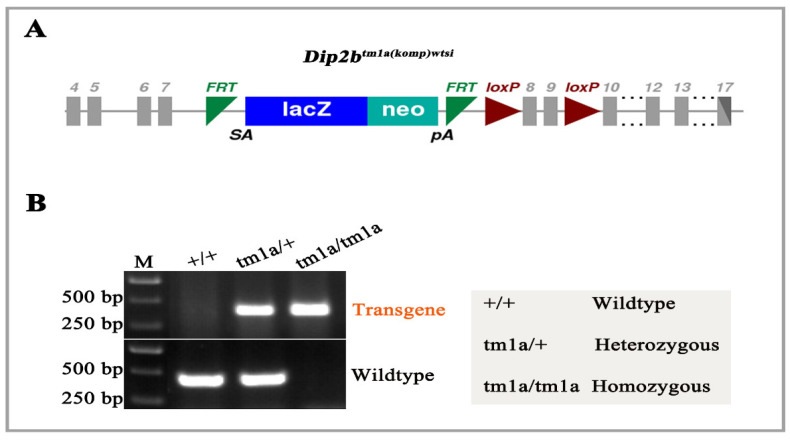
Tm1a knockout-first reporter tagged insertion allele and genotyping. (**A**) Structure of *Dip2b^tm1a (KOMP) Wtsi allele^*. A critical exon is flanked by loxP sites, with FRT, lacZ, neo, and FRT elements upstream of the critical exon. (**B**) Genotyping PCR results for tail biopsy from *Dip2b^tm1a/^*^+^ intercrossing mice. A 300 bp band appeared on the gel for the transgene allele and a 350 bp band for the wild-type allele.

LacZ reporter mice have made a huge contribution to revealing gene expression patterns in vivo and in vitro. *Dip2b* is one of the genes that has never been systematically studied, neither in terms of expression nor function, although many critical biological roles have been suggested. Studying *Dip2b* expressing results on a knockout first, reporter-tagged insertion strategy involving *Dip2b^tm1a^* mice to generate a conditional and null allele. The targeted *Dip2b^tm1a (komp) wtsi^* allele carries a targeted trap “tm1a” knockout- first allele, promoterless selection cassette inserted into the seventh intron of the *Dip2b* gene consisting of an internal ribosome entry site (En2) that allows internal translation to start from ATG of the LacZ gene, splice acceptor (sA) site that provides trans-splicing of the LacZ-Neo cassette onto exon 7, and a fusion cDNA of LacZ and Neo (Figure 1A). The presence of the splice acceptor results in expression of a truncated *Dip2b* transcript leading to production of Dip2B containing only the first 311 of 2971 amino acids. Tm1a locus genotyping was performed using primers tm1a-F and tm1a-R to give a mutant specific band of 300 bp. The wild-type primers wt-F and wt-R amplify the intron 7 region and provide a product of 350 bp (Figure 1B).

### 3.2. Expression of LacZ in the Nervous System

**Figure 2:**
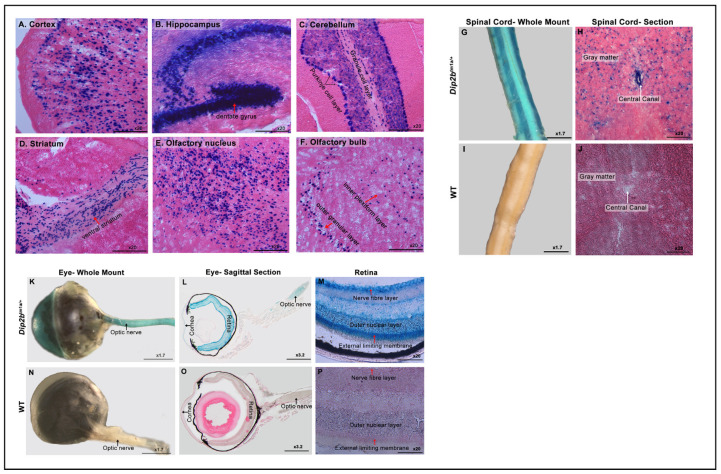
LacZ expression in the nervous system. (**A**–**F**) Frozen section expression analysis of *Dip2b^tm1a/+^* brain regions at high magnification. (**A**) LacZ protein expression is notable in cortex neurons, (**B**) the hippocampus and dentate gyrus, (**C**) the granular and Purkinje cell layer of the cerebellum, (**D)** the ventral striatum, (**E**) the olfactory nucleus, and (**F**) the inner plexiform and outer nucleus layer of the olfactory bulb in *Dip2b^tm1a/+^.* (**G**–**J**) LacZ staining of the adult spinal cord. (**G**) Whole-mount staining of the adult spinal cord. (**H**) Longitudinal section of the spinal cord depicting LacZ signals in the gray matter and central canal in *Dip2b^tm1a/+^*. (**I**,**J**) The wild-type whole-mounted and frozen sections were LacZ-negative. (**K**–**P**) LacZ staining in the adult eye. (**K**) Whole-mount staining of P56 mouse eyes, along with nerve fibers, showed LacZ staining. (**L**) Sagittal section of the eye stained for LacZ expression. The retinal wall and cornea showed LacZ expression in *Dip2b^tm1a/+^*. (**M**) LacZ-positive retina of *Dip2b^tm1a/+^* at high magnification showed strong signals in the external limiting membrane, outer nuclear layer, and optic nerve fiber layer. (**N**–**P**) Wild-type control was devoid of a LacZ-positive signal.

To address the activity of the regulatory region of *Dip2b* in the nervous system, whole-mount and frozen sections of the brain, spinal cord, and retina of 8-week-old heterozygous male *Dip2b^tm1a/+^* and wild-type (WT) mice were subjected to LacZ staining. Whole-mount staining of ventral and dorsal brain orientation demonstrated strong signals widely spread among the two olfactory lobes (OLs), cerebral hemisphere (CH), superior and inferior colliculus (coli; colr), pons (PON), and cerebellum (CB) (Supplementary Figure S1A,B). Control littermates were negative for any X-gal signals (Supplementary Figure S1C,D). However, a detailed expression pattern was clearly understood only after performing frozen section staining of the brain (Supplementary Figure S1E,F). LacZ staining of 25 μm frozen section revealed strong signals in the cortex-dense ii/iii layers, scattered evenly in the iv layer (Figure 2A), hippocampal Ammon’s horn (CA1, CA2, and CA3) including the dentate gyrus (Figure 2B), Purkinje cell layer and granular layer of the cerebellum (Figure 2C), ventral striatum (Figure 2D), olfactory nucleus (Figure 2E), and olfactory bulb consisting of the inner plexiform and outer glomerular layers (Figure 2F). Whole-mount staining allowed the detection of LacZ signals throughout the spinal cord in *Dip2b^tm1a/+^* (Figure 2G). A 25 μm cross-section of the *Dip2b^tm1a/+^* spinal cord revealed endogenous β-galactosidase activity on gray matter and the central canal (Figure 2H). Wild-type littermates did not show any LacZ signals when whole-mount (Figure 2I) or frozen sections (Figure 2J) were stained under the same conditions.

In the whole-mounted and frozen section LacZ staining of 8-week-old *Dip2b^tm1a/+^* eyes, LacZ was strongly expressed in the optic nerve (Figure 2K,L), the outer stratified squamous epithelial layer of the cornea, and the retina. At high magnification, an X-gal signal was clearly visible in the retinal optic nerve fiber layer, outer nuclear layer, and external limiting membrane (Figure 2M). As expected, the control littermates (WT) did not show staining (Figure 2N–P). In summary, Dip2B expression as indicated by β-galactosidase activity was dominant in nervous system-oriented tissues comprising of the brain, spinal cord, and eye.

### 3.3. LacZ Expression in the Reproductive System

**Figure 3:**
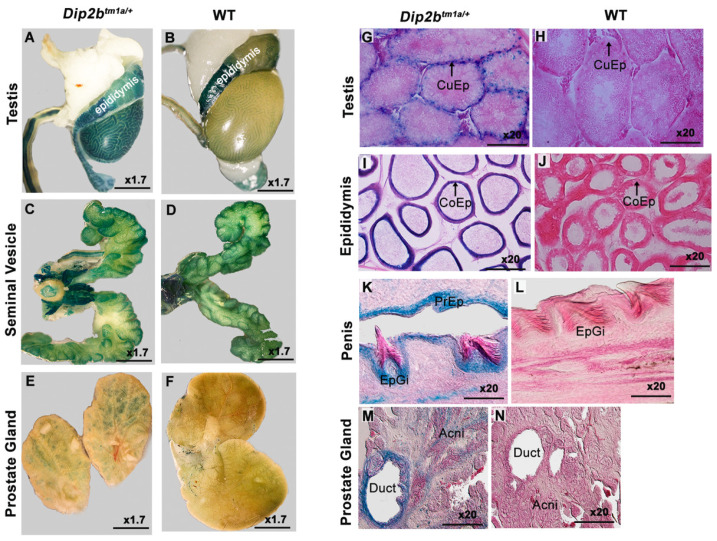
LacZ staining of the male reproductive system. (**A**–**F**) Whole-mount LacZ-stained male testis, epididymis, seminal vesicle, and prostate gland. Non-specific staining can be seen in the wild-type epididymis and *Dip2b^tm1a/+^* seminal vesicle. (**G**–**N**) Frozen section of a LacZ-stained male testis, epididymis, penis, and prostate gland. CuEp, cuboidal epithelium; CoEp, columnar epithelium; PrEp, perpetual epithelium; EpGi, glans epithelium.

Male and female reproductive organs were investigated, and LacZ expression was identified in the testes, epididymis, prostate gland, penis, vagina, ovaries, and oviduct (Figure 3). LacZ staining of the *Dip2b^tm1a/+^* male reproductive system revealed strong expression in the testes and prostate gland, while non-specific expression was observed in the epididymis and seminal vesicle (Figure 3A–F). Upon sectioning of the testes, staining was detected around the cuboidal epithelium (CuEp) lining of convoluted seminiferous tubules, which house spermatocytes, spermatids, and Sertoli and Leydig cells (Figure 3G). In the epididymis-stained section, a signal was detected at columnar epithelium (CoEp) lining of ducts (Figure 3I). In the penis section, staining was visible in the epithelium of glans and prepuce (PrEp and EpGi) (Figure 3K). In the prostate gland, staining was detected around the duct wall and acne (Figure 3M). Wild-type littermates did not show any X-gal signals upon sectioning (Figure 3H,J,L,N).

**Figure 4:**
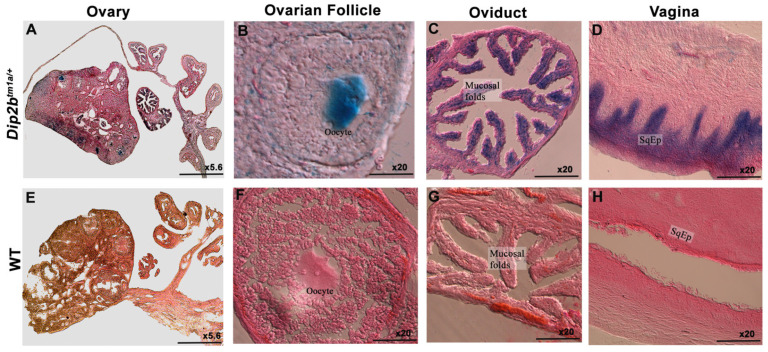
LacZ expression in the female reproductive system. (**A**) Cross-section of the ovary of a non-pregnant mouse. LacZ expression is visible in the ovarian follicles and oviduct. (**B**,**C**) High magnification of ovarian follicles and oviduct. (**D**) Section of the vagina with a LacZ signal at the stratified squamous epithelium (SqEp). (**E**–**H**) Wild-type littermate was LacZ-negative.

In the *Dip2b^tm1a/+^* female reproductive system, LacZ was strongly expressed in the ovaries, oviduct, and vagina (Figure 4). In the ovary section, a LacZ signal was detected in the oocyte cytoplasm embedded in the ovarian follicle (Figure 4A,B). In the oviduct section, staining was deposited on the columnar epithelium of the mucosal fold (Figure 4C). However, it is not clear whether the signal was restricted to epithelium only or spread along with peg cells and lamina propria. In the vagina section, staining was visible in the lining of the stratified squamous epithelium (SqEp) (Figure 4D). The wild-type tissue section did not show LacZ expression (Figure 4E–H).

### 3.4. LacZ Expression in the Digestive System

**Figure 5:**
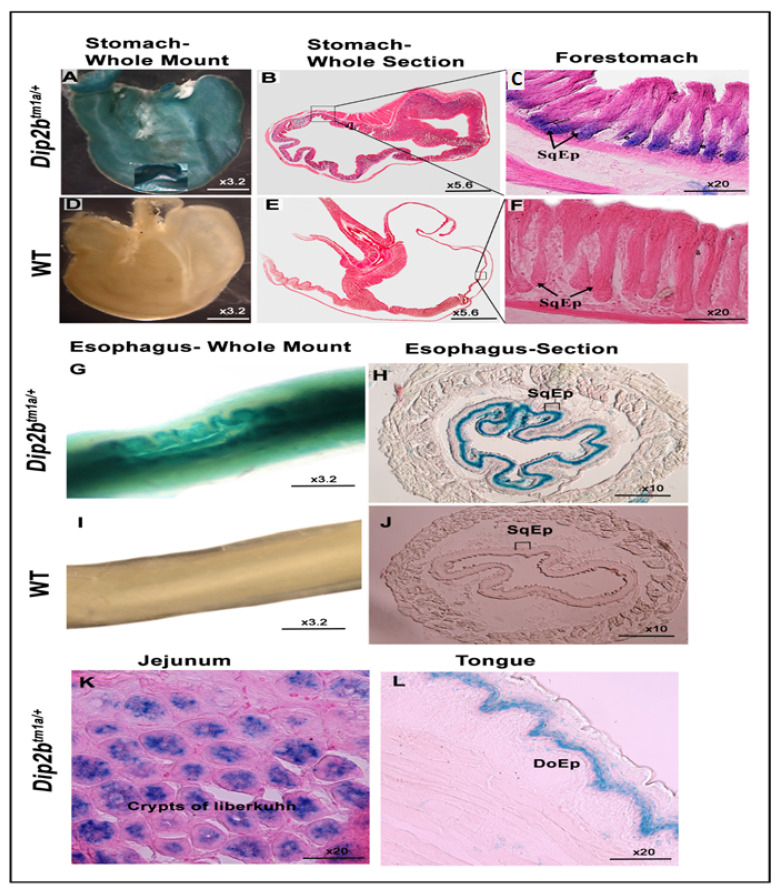
LacZ expression in the digestive system. Whole-mount and frozen section showing LacZ signals in the squamous epithelium (SqEp) of the fore stomach (**A**–**C**), esophagus (**G**,**H**), crypts of the Lieberkühn of the jejunum (**K**), and dorsal epithelium of the tongue (**L**). The control, labeled as WT, shows no staining (**D**–**F**,**I**,**J**).

After performing whole-mount and frozen tissue sectioning of the esophagus, small intestine, large intestine, and stomach, LacZ-positive signals were detected in the squamous epithelium lining of the forestomach (Figure 5A–C), in the thick mucosa layer of the esophagus comprising the non-keratinized stratified squamous epithelium (SqEp) (Figure 5G,H), in the crypts of Lieberkühn and intestinal glands in the mucosal layer of the small intestine (Figure 5K), and on the epithelial surface of the tongue (Figure 5L). Wild-type samples counterstained with LacZ were devoid of any staining (Figure 5D–F,I,J).

### 3.5. LacZ Expression of in the Respiratory System

**Figure 6:**
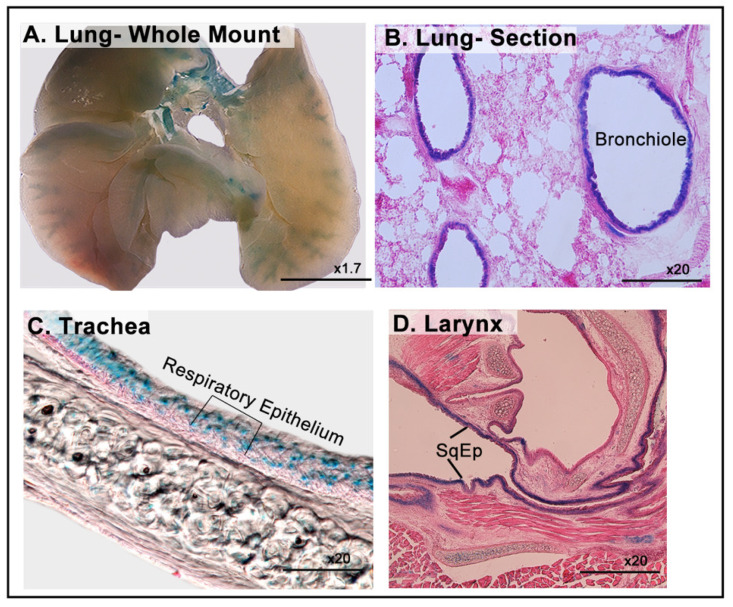
LacZ expression in the respiratory system. (**A**) Whole-mount staining of the lung. (**B**) Coronal section of the lung, showing an X-gal signal in the epithelial lining of the bronchioles. (**C**) X-gal signals in the respiratory epithelium lining of the trachea and squamous epithelium of the larynx (**D**).

Staining of *Dip2b^tm1a/+^* respiratory system comprising the lungs, trachea, and larynx were positive for β-galactosidase. In the lungs, whole-mount and frozen tissue sections showed strong and broad X-gal signals in the ciliated columnar epithelium (CoEp) lining of the bronchioles (Figure 6A,B). In the trachea, LacZ expression was strictly contained in its ciliated columnar epithelium (respiratory epithelium) (Figure 6C). Similarly, in the larynx cross-section, expression was detected in the distal stratified non-keratinizing squamous epithelium of the vocal folds (Figure 6D).

### 3.6. LacZ Expression in the Genitourinary System

**Figure 7:**
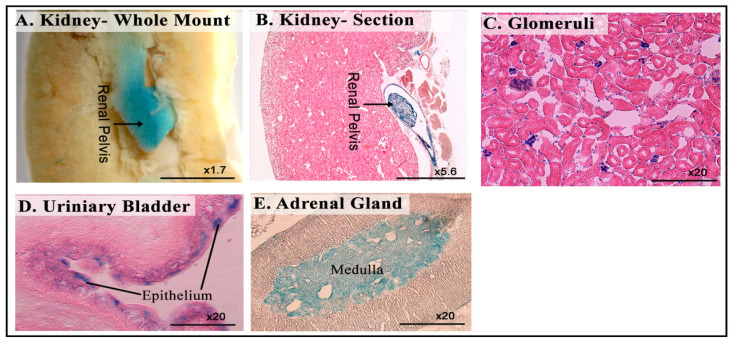
LacZ expression in the genitourinary system. (**A**–**C**) Whole-mount and frozen section LacZ staining of a kidney, showing X-gal signal at the renal pelvis and glomeruli. (**D**) Frozen section of a urinary bladder, showing LacZ signals in the epithelium. (**E**) Frozen section of an adrenal gland, showing LacZ staining in the medulla.

X-gal staining on the genitourinary system of 8-week-old *Dip2b^tm1a/+^* mice revealed strong signals in the kidneys and urinary bladder. Whole-mount and frozen tissue sections of the kidneys showed broad staining in the renal pelvis and sparse staining in the glomeruli (Figure 7A–C). In the urinary bladder, a non-specific signal was detected during the whole mount (data not shown), but after sectioning, specific staining was found in the transitional epithelium (Figure 7D). The adrenal glands showed X-gal signals strictly in the medulla, but not in the cortex (Figure 7E).

### 3.7. LacZ Expression in the Cardiovascular System

**Figure 8:**
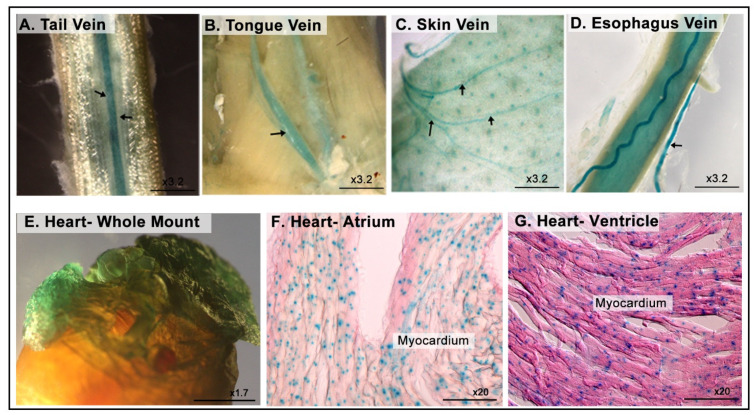
LacZ expression in the cardiovascular system. (**A**–**D**) Whole-mount LacZ staining of the veins collected from the tail, tongue, skin, and esophagus. (**E**–**G**) LacZ staining in the heart, showing uniform staining at the atrium and ventricle myocardium.

LacZ is strongly expressed in the circulatory system. Figure 8A–D shows whole-mount staining of the vascular network collected from different parts of *Dip2b^tm1a/+^* adult mice, including the tail, tongue, skin, and esophagus veins. Whole-mount and frozen tissue sections of the heart revealed LacZ signals in the atrium and the tunica media layer of ventricles (myocardium). However, it is not clear whether the signal is restricted to cardiac muscle fibers or endomysial connective tissue that contain capillaries (Figure 8E–G).

## 4. Discussion

**Figure 9:**
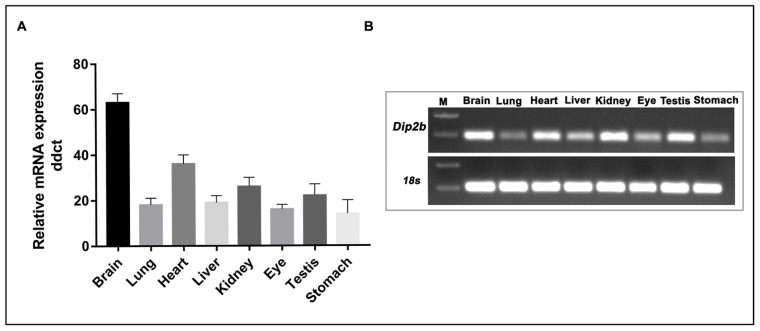
qPCR results for total RNA of 8-week-old tissues. (**A**) Relative mRNA levels were calculated using the ddCt method. Data shown are representative of at least three independent experiments and expressed as the mean ± SD. (**B**) Confirmation of the specificity of product produced by qPCR using gel electrophoresis.

In this study, *Dip2b* expression in adult tissues was investigated using Dip2b–LacZ reporter mice. The results showed that the *Dip2b* gene is widely expressed in the tissues comprising neuronal and non-neuronal cell types. The expression of *Dip2b* in various tissues was validated by real-time quantitative PCR (qPCR) and the results showed high correlation with those from LacZ staining of the corresponding tissues (Figure 9). Hence, the results of LacZ expression faithfully reflect endogenous *Dip2b* expression (Table 1).

Although the functional role of the DIP2 protein in the nervous system has been characterized in *Drosophila*, its role in the nervous systems of mammals remains to be characterized. In this study, we found that *Dip2b* to be highly expressed in the nervous system. In the brain, *Dip2b* expression was detected in the neuronal cells of all brain regions, including forebrain neurons (granular cell layer of the olfactory bulb, the cerebral cortex, and the dentate gyrus), the hindbrain (granule and Purkinje cells of the cerebellum), pyramidal neurons of the hippocampus, the thalamus, cerebellar nuclei, and the pons region. In spinal cord, *Dip2b* expression was detected in gray matter mostly comprising efferent neurons, projection neurons, and interneurons and in the central canal. These expression results indicate that *Dip2b* may play a role in neurogenesis. This is supported, in part, by our recent studies on *Dip2b* expression and its potential role during neurite outgrowth in *Dip2b^tm1a/tm1a^* hippocampal cultured cell [25]. A previous report suggested that *Dip2b* may play a role in mental retardation and in schizophrenia [3,12]. In addition, this finding also supports a previous report of Dip2B signaling in axon guidance and synaptic transmission [1]. Dip2B was initially identified as a binding partner of Disco in *Drosophila* by yeast two-hybrid screening. Mutation at this locus can cause abnormal neuronal connections in the visual system [6]. This phenotype was well supported in our study, in which high LacZ expression was observed in retinal neurons.

Our study showed that Dip2B is expressed in the vascular system, including the veins, arteries, and cardiomyocytes. Upon whole-mount staining, we observed LacZ expression in various veins, including the tongue, tail, and skin veins. This observation suggests the potential role of the *Dip2b* gene in vasculature. Integrative functional analysis of super-enhancer SNPs has shown *Dip2b* to be a novel functional locus for coronary artery disease [11]. The strong and universal LacZ expression in cardiomyocytes may suggest a potential role of Dip2B in heart function.

Besides the nervous and cardiovascular systems, our studies demonstrated that Dip2B is expressed in other somatic tissues, including the reproductive, respiratory, genitourinary, and digestive systems. Strong expression of Dip2B was detected in the epithelial cells within the testes, epididymis, penis, urinary bladder, oviduct, vagina, bronchioles, trachea, tongue, esophagus, and forestomach, which suggests the possible role of Dip2B in epithelial cells. It has been reported that Dip2B may function as an epigenetic regulator responsible for the expansion of epithelial KIT^+^ progenitor during organogenesis [26]. In addition, bioinformatics analysis has implicated *Dip2b* as a target gene in the metastasis of cervical squamous cell carcinoma [13]. LacZ expression was also detected in the prostate gland, oocytes, kidneys, adrenal glands, and intestinal glands. Cytological investigations are ongoing to identify the cell type-specific distribution of *Dip2b* gene expression and to elucidate possible functions of the *Dip2b* gene in these organs [16,25].

## 5. Conclusions

We investigated endogenous *Dip2b* gene expression in a *Dip2^tm1a^* mouse model containing the LacZ reporter gene. We found that Dip2B is expressed in a variety of somatic tissues, including the brain, eyes, spinal cord, testes, epididymis, penis, prostate gland, vagina, ovaries, oviduct, heart, lungs, and kidneys. The results of this expression study will facilitate identification of the functional role(s) of Dip2B during postnatal development and in adults. In addition, the *Dip2b^tm1a^* reporter mouse line used here will be an excellent tool for future characterization of the cell lineage and fate of *Dip2b*-expressing cells during early embryonic development, as well as for future identification of the factors and functions regulated by Dip2B and its associated diseases.

## Figures and Tables

**Table 1:** Summary of expression pattern revealed by X-gal staining of *Dip2b^tm1a/+^* mice.

*Dip2b^tm1a/+^* Expressional Pattern	
Nervous system	Cortical layers
	Ammon’s horn (CA1, CA2, CA3) and dentate gyrus of Hippocampus
	Granular and purkinje cell layer of cerebellum
	Ventral Straitum
	Olfactory nucleus
	Inner plexiform and outer nuclear layer of olfactory bulb
	Spinal cord gray matter and central canal
	Optic nerve, cornea, retinal nerve fiber layer, outer nuclear layer, and external limiting membrane
Reproductive system	cuboidal epithelium (CuEp) lining of convoluted seminiferous tubules
	columnar epithelium (CoEp) lining of epididymal ducts
	Glans and prepuce epithelium of penis
	Duct wall and acne of prostrate gland
	columnar epithelium of oviduct’s mucosal fold
	stratified squamous epithelium lining of vagina
	oocyte
Digestive system	squamous epithelium lining of fore-stomach
	non-keratinized stratified squamous epithelium layer of esophagus
	Crypts of lieberkuhn of jejunum
	Dorsal epithelium lining of stomach
Respiratory system	Bronchial epithelium of lung
	Respiratory epithelium of trachea
	Squamous epithelium of larynx
Genitourinary system	Renal pelvis and glomeruli of kidney
	Epithelium lining of urinary bladder
	Medulla of adrenal gland
Cardiovascular system	Veins
	Cardiac muscle

## Supplementary Materials

The following are available online at https://www.mdpi.com/article/10.3390/cimb43020040/s1. File S1: Supp 1. *Dip2b* disco-interacting protein 2 homolog B (*Mus musculus* (house mouse)) genomic sequence. Figure S2: Supp 2. LacZ expression in the adult brain (A–D). (A–D) Whole-mount LacZ staining on the dorsal surface (from rostral to caudal), showing the LacZ signal in the olfactory lobe (OL), cerebral hemisphere (CH), colliculus (col r; col c), and cerebellum (CB), while pons (PON) is shown on the ventral surface in *Dip**2b^tm1a/+^*. (E–F) LacZ staining of a P56 adult brain sagittal section showing LacZ staining in the olfactory nucleus (ON), stratum (ST), hippocampus (HIP), and cortex (COR). (C,D,F) Wild-type showing no LacZ-positive signal.
