# Supplementary material for: Analysis of Dip2B Expression in Adult Mouse Tissues Using the LacZ Reporter Gene
Source: Curr Issues Mol Biol. 2021 Jun 30;43(2):529–42. doi: 10.3390/cimb43020040 (PMC8929063; doi:10.3390/cimb43020040)
Supplement: Supplementary file 1 [file cimb-43-00040-s001.zip › cimb-1242110-supplementary.pdf]

Dip2B Expression Analysis in Adult Tissues Using LacZ Reporter Gene

Supplementary data:

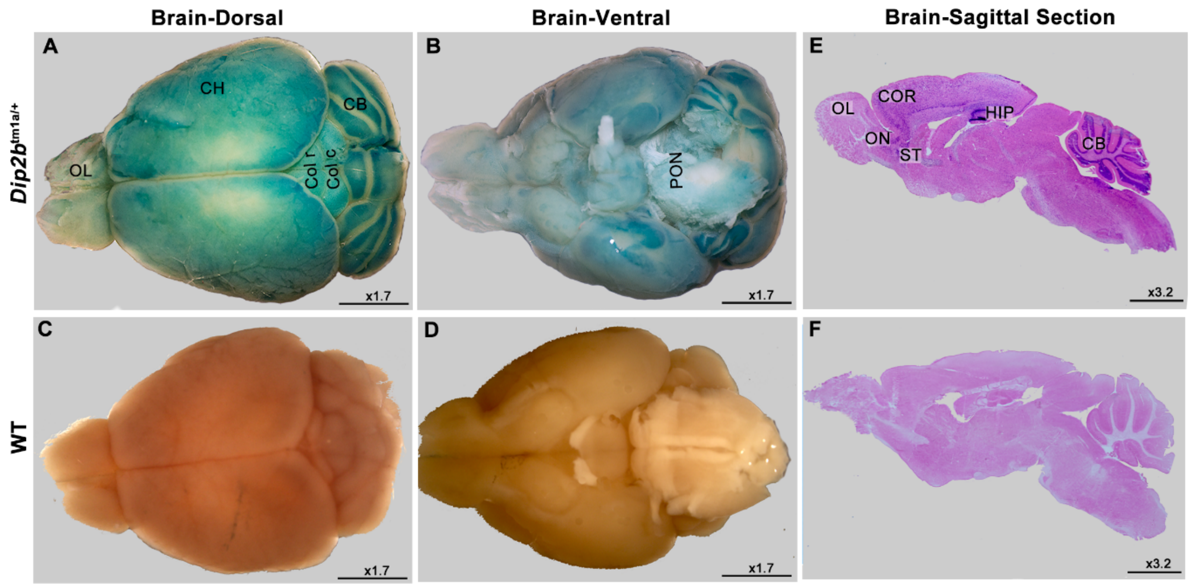

Supp 1. LacZ expression in adult brain. (A-D). **(A-D)**. Whole mount LacZ staining on the dorsal surface (from rostral to caudal), showing LacZ signal at olfactory lobe (OL), cerebral hemisphere (CH), coliculus (col r, col c), and cerebellum (CB), whereas pons (PON) on the ventral surface in *Dip2b*$^{tm1a/+}$. **(E-F)** LacZ staining of P56 adult brain sagittal section showing LacZ staining at olfactory Nucleus (ON), stratum (ST), hippocampus (HIP), and cortex (COR). **(C, D, and F)** Wild type showing no LacZ-positive signal.
